# PP2A: The Achilles Heal in MDS with 5q Deletion

**DOI:** 10.3389/fonc.2014.00264

**Published:** 2014-09-23

**Authors:** David A. Sallman, Sheng Wei, Alan List

**Affiliations:** ^1^Immunology Program and Malignant Hematology Program, H. Lee Moffitt Cancer Center and Research Institute, Tampa, FL, USA

**Keywords:** PP2A, p53, MDM2, RPS14, lenalidomide, deletion 5q, myelodysplastic syndrome

## Abstract

Myelodysplastic syndromes (MDS) represent a hematologically diverse group of myeloid neoplasms, however, one subtype characterized by an isolated deletion of chromosome 5q [del(5q)] is pathologically and clinically distinct. Patients with del(5q) MDS share biological features that account for the profound hypoplastic anemia and unique sensitivity to treatment with lenalidomide. Ineffective erythropoiesis in del(5q) MDS arises from allelic deletion of the ribosomal processing S-14 (RPS14) gene, which leads to MDM2 sequestration with consequent p53 activation and erythroid cell death. Since its approval in 2005, lenalidomide has changed the natural course of the disease. Patients who achieve transfusion independence and/or a cytogenetic response with lenalidomide have a decreased risk of progression to acute myeloid leukemia and an improved overall survival compared to non-responders. Elucidation of the mechanisms of action of lenalidomide in del(5q) MDS has advanced therapeutic strategies for this disease. The selective cytotoxicity of lenalidomide in del(5q) clones derives from inhibition of a haplodeficient phosphatase whose catalytic domain is encoded within the common deleted region on chromosome 5q, i.e., protein phosphatase 2A (PP2Acα). PP2A is a highly conserved, dual specificity phosphatase that plays an essential role in regulation of the G2/M checkpoint. Inhibition of PP2Acα results in cell-cycle arrest and apoptosis in del(5q) cells. Targeted knockdown of PP2Acα using siRNA is sufficient to sensitize non-del(5q) clones to lenalidomide. Through its inhibitory effect on PP2A, lenalidomide stabilizes MDM2 to restore p53 degradation in erythroid precursors, with subsequent arrest in G2/M. Unfortunately, the majority of patients with del(5q) MDS develop resistance to lenalidomide over time associated with PP2Acα over-expression. Targeted inhibition of PP2A with a more potent inhibitor has emerged as an attractive therapeutic approach for patients with del(5q) MDS.

## Introduction

Myelodysplastic syndromes (MDS) represent both a clinical and genetically heterogeneous group of clonal hematopoietic stem cell disorders characterized by progressive cytopenias, dysplasia, and risk of transformation into acute myeloid leukemia (AML). Cytogenetic characterization of MDS is a cornerstone of prognostic assessment for overall survival and risk of AML evolution. Conventional cytogenetic data are incorporated into both the International Prognostic Scoring System (IPSS) and the revised-IPSS (R-IPSS) (1, 2). However, chromosomal abnormalities are only found in 52% of patients (3). Over recent years, there has been considerable progress in the molecular characterization of MDS with somatic mutations found in 80% or more of cases (4). The molecular characterization of MDS adds further prognostic information with mutations involving *TP53, EZH2, ETV6, RUNX1*, and *ASXL1* associated with decreased overall survival and higher risk of AML transformation (5, 6).

Myelodysplastic syndromes with isolated chromosome 5q deletion [del(5q)] represents a distinct clinical and pathological entity recognized in the World Health Organization (WHO) classification. An interstitial deletion involving chromosome 5q is the most common cytogenetic abnormality in MDS, accounting for approximately 15% of MDS cases (3, 7). Of these, 50% have isolated del(5q) while the remaining have either an additional cytogenetic abnormality or a complex karyotype (3, 8). Del(5q) MDS is characterized by hypoproliferative anemia with dysplastic megakaryocytes and a rather indolent clinical course (9). Red blood cell (RBC) transfusion dependence develops early in the disease course and represents the principle driver of morbidity and mortality. With the advent of exome sequencing, molecular profiling has shown that 20% of patients with isolated del(5q) MDS and 72% of patients with del(5q) accompanying a complex karyotype harbor *TP53* gene mutations (7). This genetic heterogeneity accounts for the prognostic heterogeneity in clinical course and treatment outcome (8, 10). The following review is structured chronologically as each major breakthrough in the field has further defined the phenotype of del(5q) MDS and the critical role of PP2A in the treatment of these patients. Together, these data highlight novel therapeutic strategies in order to target the underlying pathogenesis of this disease.

## Lenalidomide and DEL(5q) MDS

Lenalidomide represents the first therapeutic agent in MDS, which targets a cytogenetically defined disease subset. The initial evidence of its clinical activity was based on a high clinical and cytogenetic response rate in del(5q) MDS patients in the initial safety and efficacy study (11). Lenalidomide was approved by the Food and Drug Administration (FDA) in 2005 for the treatment of transfusion-dependent IPSS low or intermediate (int)-1 risk, del(5q) MDS. The approval was based on results of the MDS-003 multicenter phase 2 trial in which 67% of patients achieved transfusion independence (TI) with lenalidomide therapy with a median TI duration of 2.2 years (12). In addition, 73% of patients had at least a partial cytogenetic response with 45% of patients achieving a complete response (CR). A recently published long-term follow up of this study found that the median overall survival was significantly increased in patients who reached TI, 4.3 vs. 2 years in non-responders, and in cytogenetic responders, 4.9 vs. 3.1 years, respectively (13). Achievement of TI or cytogenetic response also led to a decreased risk of progression to AML.

## DEL(5q) MDS Pathobiology

The initial molecular understanding stemmed from genetic mapping experiments that identified two common deleted regions (CDRs) in the 5q− syndrome (14, 15). The 5q− syndrome is characterized by isolated del(5q), female predominance, refractory macrocytic anemia accompanied by erythroid hypoplasia, normal to increased platelet counts, normal to mildly decreased neutrophil counts, and hypolobated micromegakaryocytes (16, 17). In patients with MDS or AML (excluding patients with 5q− syndrome), Horrigan et al. (15) identified a CDR at 5q31 (proximal CDR) with a minimal deletion of 1 MB leading to presumed loss of a tumor suppressor gene(s). In 5q− syndrome patients, deletion mapping of the CDR identified a 1.5 MB interval at 5q32–33 (distal CDR) (14). The distal CDR contains 40 genes, 33 of which were found to be expressed in CD34+ cells. Based on Knudson’s two-hit hypothesis, extensive sequencing of 5q− syndrome patients was performed to identify a possible second genetic event leading to the malignant phenotype. Boultwood et al. (18) compared the transcriptome of CD34+ cells from 5q− syndrome patients to the transcriptome of CD34+ cells in healthy controls but did not identify silenced genes in the remaining allele. A recent study utilizing whole-exome sequencing on del(5q) patients at baseline and at the time of leukemic transformation also found no mutations in the residual alleles of the CDR (19).

Despite the absence of identifiable mutations to account for the second-hit, expression of genes encoded within the CDR was consistent with monoallelic expression (18). Of the candidate genes, haploinsufficiency was evident in secreted protein acidic and rich in cysteine (*SPARC*), a tumor suppressor gene, and ribosomal processing S14 gene [*RPS14* (component of 40S ribosomal subunit)]. *RPS14* haploinsufficiency was of particular interest given that *RPS19* mutations represent the most common genetic mutation in patients with Diamond–Blackfan anemia (DBA), a congenital bone marrow failure syndrome with profound erythroid hypoplasia (20). Furthermore, Gazda et al. (21) had shown that haploinsufficiency of *RPS19* was the underlying defect in a proportion of patients with DBA (21). To determine the possible role of RPS14 haploinsufficiency in the pathogenesis of del(5q) MDS, Ebert and colleagues (22) designed lentiviral expressed short hairpin RNA (shRNA) as an RNA interference screen against each of the 40 genes identified in the CDR (22). Utilizing this novel methodology, only shRNA to *RPS14* re-capitulated erythroid features of the 5q− phenotype, producing erythroid maturation arrest and impaired viability (22). *RPS14* over-expression, in contrast, selectively rescued erythropoiesis in CD34+ cells of del(5q) patients but not in non-del(5q) patients. Of importance, shRNA to *RPS14* down-regulated gene expression by 60%, comparable to the haploinsufficient state of del(5q) patients. Furthermore, *RPS14* haploinsufficiency was confirmed as the underlying genetic abnormality underlying the hypoplastic anemia as *RPS14* gene sequencing in MDS patients showed absence of point mutations, biallelic deletion, or epigenetic modification (i.e., methylation) (22).

The question of how *RPS14* haploinsuffiency led to such a dramatic deleterious effect on erythroid maturation without negative consequences on myeloid or megakaryocyte lineages has recently been elucidated. Specifically, *RPS14* haploinsufficiency (via lentiviral shRNA) induced p53 accumulation in erythroid precursors and subsequent cell-cycle arrest in a lineage specific fashion (23). Impaired ribosome biogenesis as a result of RPS14 deficiency liberates free ribosomal proteins (RP) in the nucleoplasm, particularly RPL11, that bind to and sequester the human homolog of the E3 ubiquitin ligase, mouse double minute 2 protein (MDM2), the major negative regulator of p53 (24). Degradation of MDM2 drives p53 stabilization and activation (25). Interference with the assembly of the 40S ribosomal subunit, as occurs in RPS14 haploinsufficiency, induces up-regulation of the RPL11 transcript (23, 24). Treatment of CD34+ cells with Nutlin-3, a direct inhibitor of MDM2–p53 interaction (26), leads to p53 accumulation and cell death primarily in the erythroid lineage, providing a mechanistic rationale for the profound anemia in del(5q) MDS (23). These data were reproduced *in vivo* and occurred independent of ribosomal dysfunction, further supporting heightened sensitivity to p53-mediated apoptosis in erythroid precursors. Inhibition of p53 activity, via pifithrin-α, abrogates this pathway, restoring normal erythropoiesis. Peller and colleagues (27) had previously described p53 activation as a critical molecular event involved in normal erythroid maturation. The additional hit of ribosomal haploinsufficiency leading to p53 up-regulation in combination with a heightened baseline activation of the p53 pathway in erythroid precursors provide strong biological rationale for the erythroid phenotype in del(5q) MDS.

In addition to significant macrocytic anemia, del(5q) patients commonly have moderate neutropenia and thrombocytosis. The mechanism underlying these hematological features has been ascribed to deficiency of two microRNAs (miRNAs) encoded in the distal CDR, *miR-145* and *miR-146a* (28). Targeted knockdown of these miRNAs in CD34+ cells leads to a pro-inflammatory state with activation of tumor necrosis factor receptor-associated factor-6 (TRAF6), inducing IL-6 and subsequent neutropenia, thrombocytosis, and the dysplastic megakaryocytes characteristic of del(5q) MDS (28, 29). Furthermore, down-regulation of *miR-145* induces *Fli-1*, a megakaryocyte and erythroid transcription factor, accompanied by thrombocytosis (30). Combined knockdown of *RPS14* and *miR-145* was sufficient to re-capitulate the hematologic and pathologic phenotype of del(5q) MDS (30).

## Lenalidomide Selective Cytotoxicity in DEL(5q) MDS Results from Inhibition of Haplodeficient Phosphatases

**Figure 1:**
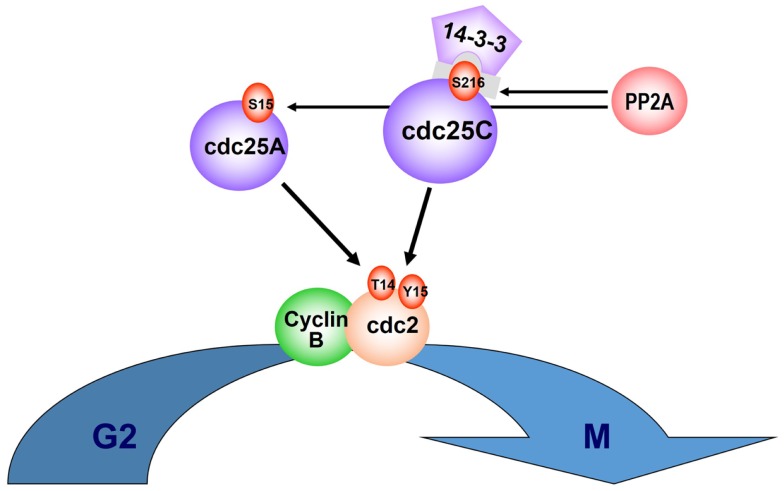
**CDC25C and PP2Acα regulatory role on cell cycle**. Entry into mitosis is mediated via PP2A dephosphorylation of Cdc25C, at the regulatory serine substrate (Ser^216^), leading to 14-3-3 displacement and nuclear translocation of the CyclinB/cdc2 complex. PP2A also regulates phosphorylation of Cdc25A at Ser^15^. Phosphorylation of tyrosine (Tyr^15^) and threonine (Thr^14^) residues within the ATP binding domain of cyclin-dependent kinase-1 (CDK-1, or cdc2) inhibit CDK-1/Cyclin B complexes and cell-cycle progression from G_2_ to M.

Just as allelic haplodeficiency of specific genes accounts for the del(5q) MDS phenotype, gene dosage of two dual specificity phosphatases encoded within or adjacent to the proximal CDR at 5q31*, CDC25C*, and *PP2Ac*α, underlies the selective suppression of del(5q) clones by lenalidomide (31). Both of these phosphatases play a pivotal co-regulatory role at the G_2_M checkpoint (32, 33). Phosphorylation of tyrosine (Tyr^15^) and threonine (Thr^14^) residues within the ATP binding domain of cyclin-dependent kinase-1 (CDK-1 or cdc2) inhibit CDK-1/Cyclin B complexes, thereby blocking cell-cycle progression from G_2_ to M. Entry into mitosis is mediated through PP2A dephosphorylation of Cdc25C at the regulatory serine substrate (Ser^216^), leading to dissociation from the 14-3-3 binding protein and nuclear translocation of the Cyclin B/cdc2 complex (34, 35). The above regulatory pathway is depicted in Figure 1. Del(5q) cells in MDS or AML patients were demonstrated to be haplodeficient for *PP2Ac*α and *CDC25C*. Lenalidomide-induced apoptosis in a concentration-dependent fashion in del(5q) AML cells while demonstrating no apoptotic effects in non-del(5q) patient cells or non-del(5q) cell lines (31). Lenalidomide was shown to inhibit the activity of Cdc25C and PP2Acα (as evidenced by retention of critical phosphate residues) by direct and indirect mechanisms, respectively. To determine if inhibition of these phosphatases is directly responsible for the cytoselective toxicity of lenalidomide in del(5q) MDS, knockdown of *CDC25C* and *PP2c*α using lentiviral shRNA was performed in non-del(5q) cells to levels commensurate with haplodeficiency. Down-regulation of *CDC25C* or *PP2c*α markedly increased sensitivity to lenalidomide, and the pro-apoptotic effects were additive with the dual gene knockdown. Of greater importance, these findings were replicated in non-del(5q) MDS bone marrow mononuclear cells (MNC) with a threefold increase in apoptosis and G2/M cell-cycle arrest.

## PP2Acα: Critical Target of Lenalidomide

Even though inhibition of the haplodeficient phosphatases Cdc25C and PP2Acα can alone account for the selective cytotoxicity of lenalidomide in del(5q) cells, how this effect could be reconciled in clones arrested in G1 by p53 activation warranted further investigation (22, 31). Although cellular p53 expression is up-regulated in bone marrow erythroid precursors of del(5q) patients, lenalidomide stabilizes MDM2 to release cells from G1 arrest by modifying its phosphorylation as a result of PP2Acα inhibition (36). Specifically, in Namalwa cells harboring a del(5q) chromosomal abnormality and primary del(5q) MDS bone marrow MNC, lenalidomide induced MDM2 expression and consequent p53 degradation. Over-expression of *PP2Ac*α suppressed MDM2 induction and restored p53 stabilization upon lenalidomide treatment, indicating that expression level of PP2Acα is a key determinant of drug-induced p53 degradation.

**Figure 2:**
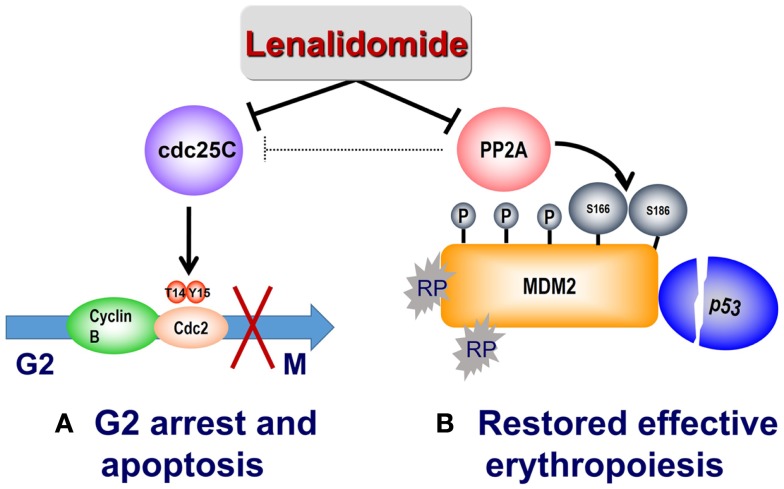
**Mechanism of action of lenalidomide in del(5q) MDS**. **(A)** Lenalidomide directly inhibits Cdc25C, resulting in G2/M arrest and apoptosis in del(5q) cells. Lenalidomide also indirectly inhibits PP2A with consequent Cdc25C inactivation. **(B)** Reduced expression of *RPS14* results in binding of free ribosomal proteins (RP) to MDM2, thereby triggering its auto-ubiquitination and p53 accumulation. Lenalidomide inhibition of the phosphatase PP2A results in hyperphosphorylation of MDM2 at Ser^166^ and Ser^186^ and stabilization of MDM2, with consequent p53 degradation and restored cell-cycle re-entry.

The RPS14–MDM2 interaction arising from nucleolar stress in del(5q) is disrupted by lenalidomide to foster transition to subsequent G2/M arrest. PP2A is also recognized to modulate p53 through dephosphorylation of Thr^55^ and Ser^46^ residues, thereby preventing proteasome targeted degradation (37, 38). Lenalidomide treatment promotes retention of the Thr^55^ and Ser^46^ phosphorylated residues with consequent down-regulation of cellular p53 (36). In addition, lenalidomide specifically increased phosphorylation at critical regulatory sites at Ser^166^ and Ser^186^, inhibiting auto-ubiquitination of MDM2 thereby leading to MDM2 nuclear translocation and p53 degradation. Phosphorylation of Ser^166^ and Ser^186^ occurs through the kinase activity of Akt, which is in turn regulated by PP2A (39, 40). Treatment with lenalidomide activated Akt as evidenced by increased phosphorylation at Thr^308^ (36). PP2Acα association with MDM2 increased in a dose-dependent manner after lenalidomide treatment while having no direct effects on PP2Acα mRNA or protein expression. Together, these data provide a biological rationale of lenalidomide mediated p53 down-regulation in PP2Acα-haplodeficient del(5q) erythroid precursors through PP2Acα inhibition and subsequent modulation of MDM2 phosphorylation. To further validate PP2Acα as the key regulator of MDM2 modulation by lenalidomide, lentiviral *PP2Ac*α shRNA were infected into non-del(5q) U937 cells (36). Whereas knockdown of *PP2Ac*α stabilized MDM2, *CDC25c* down-regulation had no effect on MDM2 expression. Similarly, decreased *MDM2* expression using siRNA prevents lenalidomide-mediated p53 degradation. The mechanism of lenalidomide’s dual actions to suppress del(5q) clones while restoring and promoting normal erythropoiesis is illustrated in Figure 2.

## Lenalidomide Resistance in DEL(5q) MDS is Associated with PP2Acα Over-Expression

PP2Acα and p53 expression in bone marrow trephine biopsies were analyzed prior to therapy, at the time of TI/cytogenetic response and at the time of treatment failure in a series of 22 del(5q) MDS patients treated with lenalidomide (36). Mean cellular expression of p53 and PP2Acα significantly decreased with treatment response. More importantly, at the time of treatment failure, PP2Acα was markedly up-regulated with restored expression of p53 in erythroid precursors, supporting our above *in vitro* data that PP2Acα over expression promotes resistance to lenalidomide. PP2Acα up-regulation in lenalidomide-resistant patients arose from a threefold increase in expression of the residual allele with no evidence for gene amplification and/or mutations in *PP2Ac*α. Change in cellular PP2Acα expression emerged as a biomarker predictive for quality of response to lenalidomide, with the magnitude of suppression at treatment response correlating with duration of TI.

Primary resistance to lenalidomide has been linked to the presence of *TP53* mutations in lower risk del(5q) MDS (41, 42). In an analysis of the French and Spanish compassionate treatment programs involving a total of 107 patients, cytogenetic response to lenalidomide in del(5q) MDS ranged from 0 to 12% in patients with mutated *TP53* compared to 73% in patients with wild-type (WT) *TP53* (41–43). Similarly, all patients who experienced a cytogenetic response also achieved TI. Whereas 73% of patients with WT-*TP53* achieved TI, only 43% of patients with mutated *TP53* achieved TI. These findings have important clinical implications as recent studies indicate that *TP53* gene mutations are demonstrable by deep sequencing in 18–20% of patients with isolated del(5q), and in 70–100% in del(5q) in combination with aberrations of chromosome 7 and/or 17 (7, 43). Moreover, ability to reach TI and cytogenetic response are directly related to OS and risk of AML transformation in patients treated with lenalidomide (13). Molecular stratification of MDS patients by *TP53* mutation status has shown decreased survival in patients harboring a *TP53* mutant clone (5), indicating that this is a critical prognostic marker that is also predictive for potential benefit from lenalidomide treatment. To this end, Saft and colleagues (44) recently reported a retrospective study of the prognostic significance of cellular p53 expression detected by immunohistochemical staining and *TP53 gene* mutation in a series of IPSS low or int-1 risk del(5q) MDS patients treated with lenalidomide in the MDS-004 trial (44, 45). Strong cellular p53 expression by IHC in ≥1% of bone marrow precursors strongly correlated with decreased cytogenetic response (*P* = 0.009), higher risk of leukemic transformation (*P* = 0.00006) and shorter overall survival (*P* = 0.0175) (44). Strong p53 positivity directly correlated with *TP53* mutation whereas moderate positivity correlated with WT-*TP53*. Presence of strong p53 staining was the most important prognostic co-variate for risk of leukemic transformation, which accounts in part for the apparent heterogeneity of risk in isolated del(5q) patients. This was exemplified by the MDS-004 trial where 3-year OS and AML transformation rates were 56 and 25%, respectively, which correlates with high percentage of strongly p53+ patients in this trial (35%) (44, 45). Furthermore, sequential whole-exome sequencing of two del(5q) patients at baseline and at AML transformation identified *TP53* mutations in both patients at the time of leukemic transformation [but not in two non-del(5q) patients], suggesting that *TP53* mutation is the critical event in progression of del(5q) MDS (19). The precise reason for the disproportionate prevalence of *TP53* gene mutations in del(5q) MDS is unknown, but one could speculate that mutant clones may be selected for under the pressure of constitutive activation of this pathway in this particular cytogenetic MDS subset, analogous to other cancers in which such mutations emerge in the presence of ongoing cellular stress or DNA-damage response activation (46). Targeting the RPS14–MDM2–p53 pathway by PP2A inhibition or alternative therapeutic strategies could prove to alter the natural course of the disease by preventing clonal evolution and transformation to leukemia. As sustained suppression of p53 would lead to genomic instability (46), careful clinical investigation needs to be performed to balance clinical benefit with potential risk.

## Novel Therapeutic Strategies for DEL(5q) MDS

**Figure 3:**
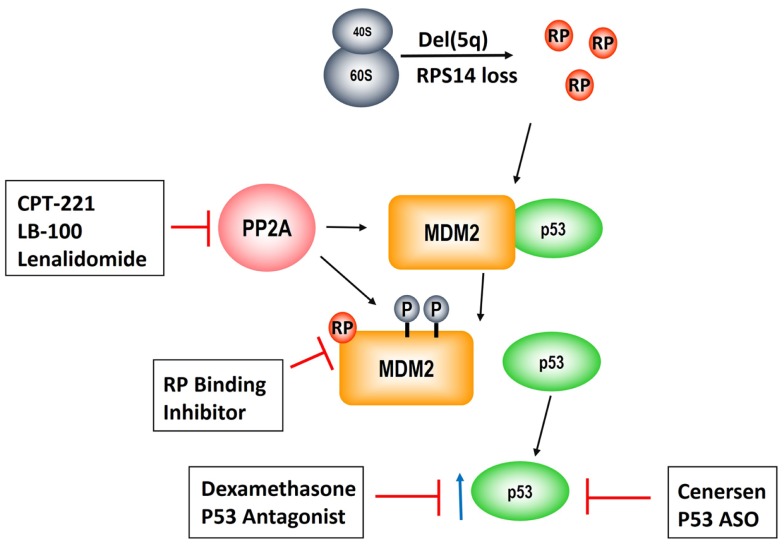
**Pathogenesis of the del(5q) MDS hematologic phenotype and novel therapeutic strategies to rescue erythropoiesis**. Allelic deletion of the genes encoded in the del(5q) CDR disrupts ribosomal integrity via haploinsufficiency of *RPS14* leading to RP, e.g., RPL11, sequestration of MDM2 and consequent p53 activation. PP2A is a negative regulatory phosphatase of MDM2 whose inhibition results in hyperphosphorylation at phosphate residues that protect it from ubiquitination. Lenalidomide abrogates the anemia in del(5q) MDS through PP2A inhibition. CP2-221 is a deuterium-modified analog of *S*-lenalidomide enantiomer with greater potency than the parent compound. LB-100 is a novel direct PP2A inhibitor that has entered clinical testing. Lastly, p53 modulation via dexamethasone and/or cenersen (p53 antisense) are exciting potential clinical strategies that are currently under clinical investigation.

As described above, PP2Acα is a key target of lenalidomide leading to modulation of the RP–MDM2–P53 pathway, clonal suppression, and restoration of normal erythropoiesis. Unfortunately, approximately 50% of patients develop resistance to lenalidomide after 2–3 years of treatment and there are currently no alternative karyotype selective therapeutic agents (13, 47). Given that *PP2Ac*α over-expression underlies lenalidomide resistance, novel strategies targeting this pathway are of pivotal importance (Figure 3). This is reinforced by our findings that duration of TI to lenalidomide was directly related to the magnitude of PP2Acα suppression (36). Specifically, median duration of TI was not reached in patients with PP2Acα suppression from baseline (1507+ days) versus 679 days in patients without (*P* = 0.006, log rank).

One potential strategy is development of more potent and specific inhibitors of PP2A. Okadaic acid, a derivative of shellfish toxin, inhibits both PP2A and PP1 (48). LB-100 and LB-102 are synthetic derivatives of cantharadin, a demethylated homolog of cantharadin (extract of beetle juice), which is an inhibitor of PP2A with relative specificity *in vitro* and *in vivo* and acceptable toxicity (49–52). LB-102 was shown to increase Akt phosphorylation and decrease p53 expression in malignant glioma cells and xenografts (50). LB-100/LB-102 blocked cell-cycle arrest and led to chemotherapy sensitization to temozolomide and doxorubicin (50, 52). LB-100 was also shown to induce tumor differentiation and/or cell death in glioblastoma multiforme (53). LB-100 has now entered a phase 1 clinical trial as a chemotherapy sensitizer in solid tumors (NCT01837667) (54). A phase 1 clinical trial of LB-100 is planned in lower risk MDS patients with symptomatic anemia, including del(5q) MDS patients who are resistant and/or intolerant to lenalidomide. This would be a first in class novel inhibitor as there are currently no FDA approved phosphatase inhibitors. Another potential strategy to enhance PP2Acα inhibition would be using the lenalidomide analog, CPT-221. CP2-221 is a deuterium-modified analog of *S*-lenalidomide (lenalidomide is a racemic mixture of S- and R-enantiomers). *In vitro* studies have shown that CPT-221 is threefold to fivefold more potent than the lenalidomide racemic mixture (55). Investigation of CPT-221 in the treatment of MDS patients has not yet been initiated.

Given that p53 accumulation in erythroid precursors is responsible for the hypoplastic anemia in del(5q) MDS, targeting p53 directly could restore terminal differentiation and effective erythropoiesis in the del(5q) clone. Cenersen, a clinically active 20-mer antisense phosphorothioate oligonucleotide complementary to exon 10, down-regulates both WT and mutant *TP53* expression *in vitro* and *in vivo* when administered in combination with chemotherapy in patients with hematologic malignancies (56–58). Cenersen treatment of *RPS14* deficient erythroblasts, to simulate the haplodeficient state in del(5q) cells, decreased cellular expression of *TP53*, leading to improved cell survival (59). Treatment of bone marrow CD34+ cells from lenalidomide resistant, del(5q) MDS with cenersen dramatically increased erythroid colony formation with less improvement in non-del(5q) or normal controls. Of interest, cenersen had no suppressive effect on the del(5q) clone, thereby supporting the notion that p53 suppression restored erythropoietic potential within the malignant clone (Figure 2). A phase 1 clinical trial testing cenersen in del(5q) MDS patients resistant and/or intolerant to lenalidomide therapy is now underway. As proof of this biological principle, Caceres et al. (59) tested the addition of dexamethasone, a glucocorticoid-receptor-dependent p53 antagonist, to lenalidomide treatment in del(5q) MDS patients who acquired resistance to lenalidomide. In this pilot study, 20 mg of weekly dexamethasone down-regulated p53 expression in erythroid progenitors and consequently restored TI in five of eight patients. The potential to achieve targeted suppression of WT and mutant *TP53* with cenersen is exciting, given the decreased efficacy of lenalidomide in patients with a *TP53* mutation (41–43). Of particular interest, Woll and colleagues (60) recently discovered that acquired driver mutations in the malignant stem cells of del(5q) MDS patients, especially *TP53*, emerge prior to leukemic transformation. This builds on prior work, which also showed that chromosome 5q deletion is the initial inciting event and is present in both the progenitor and malignant stem cells of these patients (60, 61). These stem cells that harbor del(5q) are intrinsically resistant to lenalidomide secondary to their quiescent state (61). Acquisition of *TP53* mutations could foster propagation of myeloid progenitor cells leading to hematologic relapse and disease progression in these patients. Targeting of WT and mutated *TP53* could prove to be a promising therapeutic strategy in possibly achieving and/or prolonging remissions in del(5q) MDS patients treated with lenalidomide.

## Conclusion

Myelodysplastic syndromes with isolated del(5q) represents a distinct disease subtype distinguished by clinical presentation and unique sensitivity to lenalidomide. These pathognomonic features are directly linked to haplodeficiency of critical targets in the CDR on chromosome 5q. Severe anemia constitutes the major morbidity/mortality for del(5q) MDS patients and arises from *RPS14* haplodeficiency leading to MDM2 sequestration, p53 stabilization, and erythroid cell death. Lenalidomide inhibits this pathway through its inhibitory effect on PP2A, rescuing erythropoiesis. Lenalidomide’s selective cytotoxity to del(5q) cells arises from inhibition of two haplodeficient phosphatases, PP2Acα and Cdc25C. Clonal suppression of del(5q) is of pivotal importance in preventing leukemic transformation and death. However, resistance to treatment emerges over time and future studies targeting PP2A and/or the RP–MDM2–p53 axis should be a priority to improve outcomes in this patient population.

## Conflict of Interest Statement

Dr. David A. Sallman and Dr. Alan List have patent pending for PP2A inhibitor in MDS. Dr. Alan List is a consultant for Celgene. Dr. Sheng Wei has no conflict of interest
